# A comparison and evaluation of the use of three orthodontic indices in assessing the treatment outcome+

**DOI:** 10.2340/aos.v85.46998

**Published:** 2026-10-15

**Authors:** Rayed Aldausari, Maryam Al-Zayer, Marie Pegelow

**Affiliations:** a Department of Orthodontics, Faculty of Dentistry, King Abdulaziz University, Jeddah, Saudi Arabia; b Department of Dental Medicine, Division of Orthodontics, Karolinska Institutet, Stockholm, Sweden; c Orthodontist, Dr. Zaki Dental Clinics, Al-Qatif, Saudi Arabia

**Keywords:** IOTN, ICON, PAR, orthodontic treatment, occlusal indices, treatment outcome

## Abstract

**Objective:**

To assess the differences in treatment outcome evaluation using the Dental Health Component (DHC) and Aesthetic Component (AC) of the Index of Orthodontic Treatment Need (IOTN), the Index of Complexity, Outcome and Need (ICON), and the Peer Assessment Rating (PAR), and to examine scoring agreement between orthodontic residents and undergraduate students.

**Material and methods:**

A total of 87 study casts were assessed using the IOTN, ICON, and PAR indices to evaluate treatment outcome, with intra- and inter-examiner reliability examined.

**Results:**

High to very high correlation was observed among AC, ICON, and PAR at T1 (*p* < 0.0001), while at T2, a high correlation was found between ICON and AC (*p* < 0.0001). Low correlations were found between DHC and both PAR and ICON. A negligible correlation was found between AC and DHC at T2. IOTN components showed good reproducibility at T1, whereas DHC, ICON, and PAR demonstrated moderate reliability at T2. Among orthodontic residents, ICON showed moderate (T1) and good (T2) reliability. PAR showed excellent reliability at T1. Among all examiners, all indices had moderate reliability at T1, and both ICON and PAR had poor reliability at T2.

**Conclusions:**

ICON was the most reliable index among orthodontic residents and required less scoring time than PAR, although IOTN remained the quickest to apply.

## Introduction

Malocclusion refers to a deviation from normal occlusion with aesthetic and functional implications [1]. Orthodontic treatment is justified by its potential to improve dental health, aesthetics, occlusal function, and psychosocial well-being [2]. However, the assessment of malocclusion and treatment need is often influenced by subjectivity and inconsistent interpretation [3]. To address this limitation, occlusal indices were introduced initially to support epidemiological investigations [4]. Earlier epidemiological studies frequently lacked standardized measurement methods, recording malocclusion traits in a dichotomous manner rather than as quantifiable variables [5]. Since then, several classification systems have been developed based on the intended purpose of assessment [6–12].

Objective assessment of treatment outcome in orthodontics is important for individual patients as well as for the assessment of overall quality of care provided by practitioners [13], and treatment outcomes can be quantified using the same indices used to measure the severity of malocclusion [5]. Treatment outcome is affected by multiple factors including initial malocclusion, case complexity and clinician experience [13]. Therefore, occlusal indices have become valuable tools in orthodontic research, audit, quality assurance and practice management as they promote examiner consistency and standardization of treatment outcome assessment [2, 14].

The Index of Orthodontic Treatment Need (IOTN) was primarily developed to assess the need and priority for orthodontic treatment [15, 16]. It can also be used to express the success of treatment or the extent of improvement following treatment in both individual patients and populations [6, 13, 17]. Several other occlusal indices have been developed to assess treatment outcomes such as the Peer Assessment Rating (PAR) index and the Index of Complexity, Outcome and Need (ICON). In 1987, the British Orthodontic Standards Working Party, after a series of meetings with 10 experienced orthodontists, developed the PAR index [18]. The aim of the PAR index was to provide a standardized objective method for quantifying malocclusion and assessing the results of orthodontic treatment by producing one summary score that reflects the extent to which a case deviates from normal alignment and occlusion [18]. The PAR index measures deviations from an ideal occlusion by allocating weighted scores to five distinct traits: upper and lower anterior alignment, buccal segment occlusion, overjet, overbite, and centerline discrepancies [13, 18]. The difference between pre-treatment and post-treatment weighted PAR scores mathematically reflects the exact degree of clinical improvement [13]. The PAR index is a valid tool for evaluating orthodontic treatment results [19]. Furthermore, it is considered one of the most commonly recognized instruments and is essential for commissioning and assessing the quality of national health service orthodontic treatment delivery [19]. It has also become universally accepted as a reliable standard for institutional quality control and evaluating treatment outcomes.

The ICON was developed based on the consensus of an international panel of 97 orthodontists from nine countries [2]. It has the advantage of unifying the evaluation of various treatment stages into a single index. The ICON is a multipurpose index uniquely designed to assess treatment need, complexity, and clinical outcome [20]. It evaluates five highly predictive occlusal traits: an aesthetic assessment (based on the IOTN Aesthetic Component [AC]), upper arch crowding or spacing, crossbite, anterior vertical relationship, and anteroposterior buccal segment relationship [2]. ICON uses specified outcome thresholds, in contrast to indices that are exclusively restricted to pre-treatment screening. Post-treatment study casts are assessed during outcome assessment, and an ultimate score of less than 31 indicates a clinically acceptable outcome [2]. The validation of ICON was assessed, and it is considered a valid index for evaluating orthodontic cases for treatment complexity and outcome [21]. ICON functions as a comprehensive quality assurance standard by assessing the initial complexity in conjunction with the final outcome through the integration of both objective measurements and subjective aesthetic judgments.

The validity and reliability of the PAR and ICON indices are well documented among experienced orthodontists. However, less is known about their reliability when applied by examiners with different levels of orthodontic training. An index demonstrating substantial variation in reliability with examiner training may be problematic for less experienced clinicians or in undergraduate education. Therefore, evaluating these indices across different levels of clinical training may provide additional information regarding their practical and educational applicability.

The orthodontic indices used in this investigation were selected from a wide range of indices in orthodontic literature. The three indices selected (IOTN, ICON, and PAR index) represent contemporary preference in the orthodontic field. The IOTN is used to assess treatment need, the PAR index is used to evaluate treatment outcome, and the ICON is used to assess both treatment need and outcome.

## Aim

The aim of the study was to compare the IOTN, the ICON and the PAR in evaluating the treatment outcome. A further purpose of this investigation was to compare the agreement between orthodontic residents and undergraduate students in scoring all three indices. We hypothesize that there is no difference between all three occlusal indices in evaluating the treatment outcomes, and the clinical training level of the examiner does not affect scoring reliability.

## Material and methods

### Study design

This was a retrospective, longitudinal, observational study for treatment outcome evaluation.

### The sample

The research was conducted in accordance with the Declaration of Helsinki II and received approval from the Regional Ethical Review Board (Dnr 2022-06075-01), Stockholm, Sweden. The material was chosen from 180 plaster study models, consecutively included, from patients who were in permanent dentition stage and had completed their orthodontic treatment and got their appliances removed. All patients were treated by six orthodontic residents in their Master program (2016–2019) at the Postgraduates Orthodontics Clinics, Department of Dental Medicine, Karolinska Institutet, Huddinge, Sweden. The inclusion criteria for the study required the availability of pre-treatment study casts obtained prior to the initiation of orthodontic treatment and post-treatment study casts taken immediately after treatment completion. All casts were intact, demonstrating good articulation and free of voids, fractures, or chipped teeth. Patients included in the study had received orthodontic treatment at the Karolinska postgraduate orthodontic clinics in Huddinge and were treated by supervised orthodontic residents using upper and lower pre-adjusted fixed orthodontic appliances. All chosen patients had completed their orthodontic treatment by 2019, and cases were included irrespective of sex, ethnicity, or type of malocclusion.

After excluding some participants due to uncompleted treatment, or due to missing, chipped, or broken dental casts, the final sample included 87 patients with two pairs of study casts (pre-treatment and post-treatment). The sample represents the full spectrum of malocclusion types of patients presented to the orthodontics department in Karolinska Institutet. No attempt was made to select the patient based on the type of malocclusion, gender, or extraction pattern. A representative example of the standards of the accepted dental casts included in the study is shown in Figure 1.

**Figure 1:**
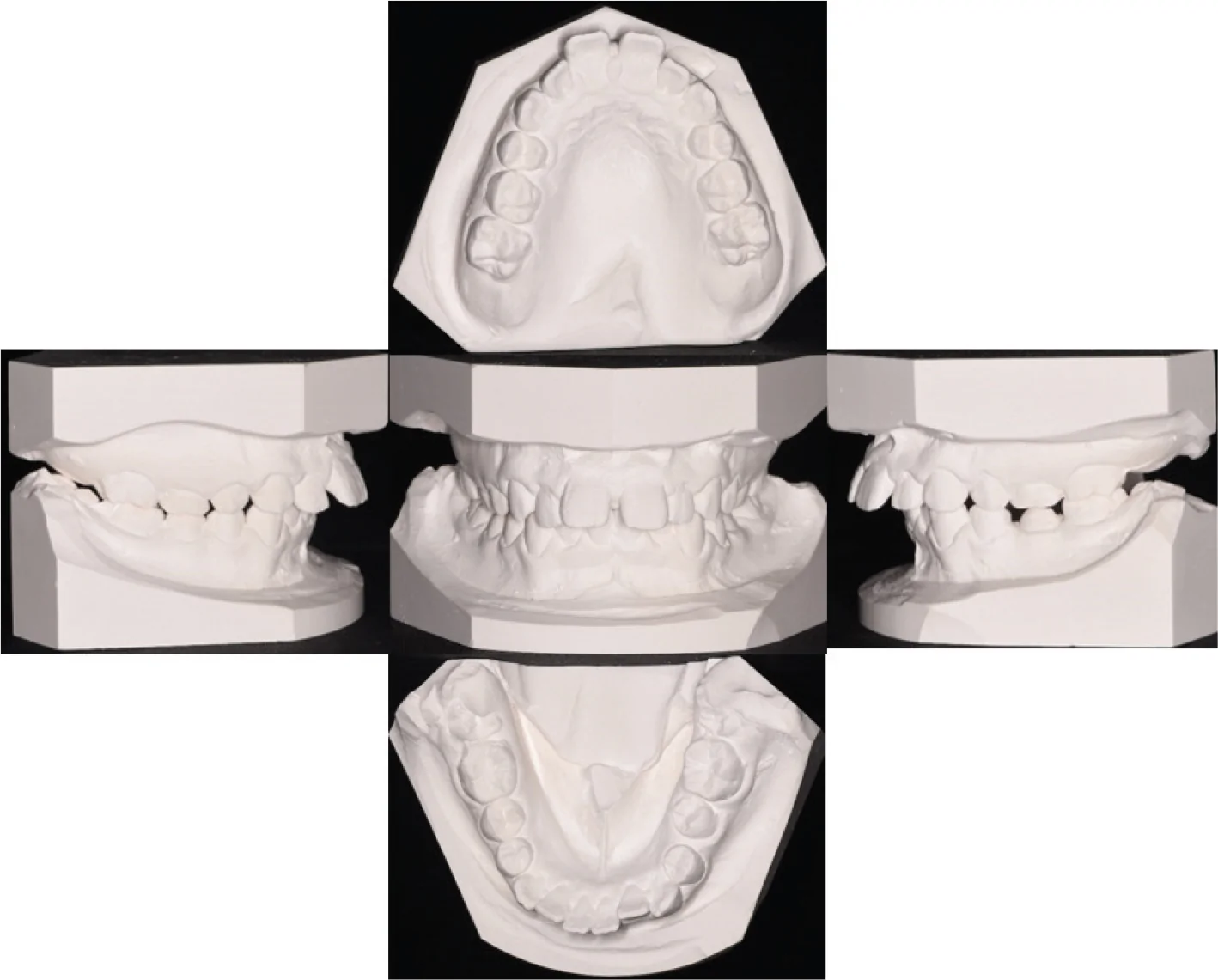
Example of the dental casts included in the study.

### Experimental design

Pre- and post-treatment study casts were examined by a calibrated examiner (MZ, a 31-year-old female postgraduate resident with approximately 3 years of clinical experience). The examiner was a postgraduate orthodontic resident at the Department of Orthodontics, Karolinska Institutet and was calibrated in three orthodontic indices namely: the IOTN, the ICON, and PAR. The Dental Health Component (DHC) of the IOTN contains five grades which include grade 1 and 2 representing ‘no or little need’, grade 3 ‘border line’ and grade 4 and 5 that represent ‘a great treatment need’. Recording the malocclusion of greatest severity of the DHC follows a hierarchical scale in descending order as follows: missing teeth, overjet, crossbite, displacement of contact points, and overbite (including open bite). The abbreviation (MOCDO) can be used to remember the hierarchical scale.The second component of the IOTN is the AC which assesses the aesthetic impairment caused by malocclusion. [16]. It consists of a scale of 10 colored intra-oral photographs showing different levels of attractiveness. The photographs are arranged from the most attractive (grade 1) to the least attractive (grade 10) appearance [6, 16].

The ICON consists of five components: aesthetic assessment based on the IOTN AC, upper arch crowding or spacing, presence of crossbite, anterior vertical relationship including open bite, and the anteroposterior relationship in the buccal segment. Each component is scored individually, and the weighted scores are summed to generate the final ICON score. Treatment need is assessed using pretreatment study casts, and orthodontic treatment is indicated when the total weighted score exceeds 43 [2]. The outcome is acceptable if the total score of the ICON is less than 31. PAR measures the occlusal changes by allocating scores to different occlusal traits including the dentition alignment, buccal segment relation, overjet, overbite and midline discrepancy which make up the occlusion [13, 18]. The individual scores are summed up to get an overall score that represents the occlusion [13, 18]. One month later, 20 of the study casts were selected randomly and re-scored by the same examiner (MZ) to evaluate the reproducibility (intra-examiner reliability) for each of the three indices. Another calibrated examiner (RD, a 32-year-old male postgraduate resident with approximately 3 years of clinical experience) who was an orthodontic resident at the department of orthodontics, Karolinska institutet, examined a randomly selected 20 pairs of dental casts to evaluate inter-examiner reliability. Two final-year undergraduate students (JH and JD, 25-year-old female students with no previous experience of using those indices) were calibrated on the three indices and evaluated the same 20 pairs to evaluate the reliability between orthodontic residents and undergraduate students. All raters scored the study casts at their own pace with no restriction of time. The time taken to score the indices per cast was recorded in minutes using stopwatch. The calibration was done with an expert orthodontist (MP) who is experienced with the three indices. The calibration consisted of a 2-hour theoretical and practical training session. Due to scheduling constraints, these calibration sessions were conducted separately for the different examiners. All assessments were performed over a time frame of 5 weeks. Treatment needs cut-off points were set as follows: the ICON 43, DHC grade 4 and 5. Due to the fact that all patients had treatment need, since they all were treated, the AC of the IOTN got the range of (5–9) in pre-treatment assessment. A score of 10 in the AC represents a very high treatment need. For the outcome assessment, IOTN of 1 and 2 indicate no treatment need which reflects a good result. The ICON describes the improvement degree in three categories (>-1 greatly improved), (-53 till -1 substantially improved or moderately improved) and (<-54 minimally or not improved). The PAR index has three categories to assess the improvement: worse or no different, improved, and greatly improved. A change of less than 22 points reduction and at least 30% PAR score reduction assigned as (improved). At least 22 points reduction assigned as (greatly improved). The assessment of the study casts was done by using appropriate examination tools and examination sheets. Written instructions for each of the three indices were followed. The data files that were generated by scoring all the study models were transferred to a spreadsheet (Microsoft Excel Sheet).

### Statistical analysis

Descriptive statistics were calculated to measure the mean and standard deviation (SD) values for DHC, AC, ICON and PAR indices. These index scores were calculated before and after treatment. However, the correlation level between all indices was assessed by the Pearson Correlation test and can be interpreted this way: 0–0.3 (negligible), 0.3–0.50 (low), 0.50–0.70 (moderate), 0.70–0.90 (high) and 0.90–1 (very high). The ICON index originally had five improvement grades, but the PAR index has three grades after treatment that made the comparison difficult. A modification was made by considering categories of the ICON index (‘minimally improved and not improved or worse’ and ‘substantially improved and moderately improved’) as two wide categories. Intra-examiner reliability was examined by repeating the examination of a subset of 20 pre-treatment and post-treatment cases by the same examiner (MZ). Intra-examiner reliability was expressed using intra-class correlation coefficients calculated for IOTN/DHC, IOTN/AC, ICON and PAR indices. The intraclass correlation coefficients (ICC) can theoretically vary between 0 and 1.0, where a value of 0 indicates no reliability and an ICC of 1.0 indicates perfect reliability. Inter-examiner reliability was assessed using ICC based on the scores of 20 randomly selected cases. Reliability was evaluated separately for the pre-treatment and post-treatment assessments. The value of *p* < 0.05 was considered statistically significant for all the correlation statistics. All the data were analyzed using the statistical analysis program SPSS (IBM-SPSS, Version 25, Armonk, NY: USA).

## Results

### Descriptive statistics

The sample consisted of 87 patients, 57.3% female and 42.7% male. The average score for each index before orthodontic treatment was as follows: DHC (mean = 4.1, SD ± 0.6), AC (mean = 7.3, SD ± 1.5), ICON (mean = 74.8, SD ± 15.1) and PAR (mean = 31.9, SD ± 12.5). After orthodontic treatment, the average score for both DHC and AC were mean = 1.4, SD ± 0.9 and mean = 1.2, SD ± 1 respectively. Furthermore, the ICON recorded mean = 12.5, SD ± 10.8 and the PAR mean = 4.2, SD ± 6 after orthodontic treatment. Descriptive statistics for all indices before (T1) and after (T2) treatment are listed in Table 1.

**Table 1:** Descriptive statistics between DHC of the IOTN, AC of IOTN, PAR, and the ICON.

Index (*n* = 87)	Mean	SD	Median
DHC (T1)	4.1	0.6	4.00
DHC (T2)	1.4	0.9	1.00
AC (T1)	7.3	1.5	8.00
AC (T2)	1.2	1.0	1.00
ICON (T1)	74.8	15.1	76.00
ICON (T2)	12.5	10.8	10.00
PAR (T1)	31.9	12.5	32.00
PAR (T2)	4.2	6	2.00

DHC: dental health component; IOTN: Index of Orthodontic Treatment Need; AC: Aesthetic component; PAR: Peer assessment rating index; ICON: Index of complexity, outcome and need; SD: standard deviation; T1: pre-treatment; T2: post-treatment.

According to the IOTN grading system, 23% of the patients analyzed were classified as IOTN grade 5 and 70.1% as grade 4 before treatment. The remaining 6.9% were classified as grade 3 and 2. However, IOTN grades were significantly reduced following orthodontic treatment. Only 2.3% of the patients treated were still in grade 5 and another 2.3% in grade 4, and 3.4% in grade 3. Moreover, the percentage of grade 2 and grade 1 significantly increased after treatment to 17.2 and 74.7 respectively.

### Correlation among the three indices

The correlation level between all indices before treatment (T1) is presented in Table 2. The results indicate that both AC and ICON had high correlation with PAR index. A very high correlation was found between ICON and AC (*p* < 0.0001). Moreover, a moderate correlation was recorded between DHC and each AC, ICON, and PAR (*p* < 0.0001). The degree of correlation between all indices after treatment (T2) is shown in Table 3. The values indicate a negligible correlation between AC and DHC (*p* = 0.015), low correlation level between DHC with both PAR and ICON (*p* = 0.002 and *p* < 0.0001 respectively), moderate correlation between PAR and both AC and ICON and high correlation level between ICON and AC (*p* < 0.0001).

**Table 2:** T1 correlations between DHC of the IOTN, AC of the IOTN, PAR, and the ICON.

Indices	DHC	AC	ICON	PAR	*p*
DHC		0.522**	0.600**	0.567**	0.000
AC	0.522**		0.930**	0.780**	0.000
ICON	0.600**	0.930**		0.832**	0.000
PAR	0.567**	0.780**	0.832**		0.000

DHC: dental health component; IOTN: Index of Orthodontic Treatment Need; AC: Aesthetic component; PAR: Peer assessment rating index; ICON: Index of complexity, outcome and need; T1: pre-treatment.

** Correlation is significant at the 0.01 level (2-tailed).

Pearsons Degree of Correlation: (0–0.3 negligible), (0.3–0.50 low), (0.50–0.70 moderate), (0.70–0.90 high) and (0.90–1 very high).

**Table 3:** T2 correlation between DHC of the IOTN, AC of IOTN, PAR, and the ICON.

Indices		DHC	AC	ICON	PAR
DHC	Correlation		0.259*	0.399**	0.331**
	*p*-value		0.015	0.000	0.002
AC	Correlation	0.259*		0.779**	0.549**
	*p*-value	0.015		0.000	0.000
ICON	Correlation	0.399**	0.779**		0.610**
	*p*-value	0.000	0.000		0.000
PAR	Correlation	0.331**	0.549**	0.610**	
	*p*-value	0.002	0.000	0.000	

DHC: dental health component; IOTN: Index of Orthodontic Treatment Need; AC: Aesthetic component; PAR: Peer assessment rating index; ICON: Index of complexity, outcome and need; SD: standard deviation; T2: post-treatment.

* Correlation is significant at the 0.05 level (2-tailed).

** Correlation is significant at the 0.01 level (2-tailed).

Pearsons Degree of Correlation: (0–0.3 negligible), (0.3–0.50 low), (0.50–0.70 moderate), (0.70–0.90 high) and (0.90–1 very high).

### Intra-examiner reliability

The results represent good reliability in scoring both DHC and AC with ICC = 0.839 and ICC = 0.801 respectively at T1. Moderate reliability in scoring both pre-treatment ICON and PAR with ICC 0.708 and 0.514 respectively. Whereas all indices DHC, ICON, and PAR at T2 show good to moderate reliability level with ICC 0.645, 0.708, and 0.517 respectively for 20 randomly selected patients who were assessed twice.

### Inter-examiner reliability

Inter-examiner reliability of randomly selected 20 patients among two orthodontic residents shows that the two residents had moderate agreement level (ICC = 0.553) in measuring DHC and poor reliability in assessing AC (ICC = 0.052) before treatment. Both examiners had moderate agreement level in scoring pre-treatment ICON (ICC = 0.538) and excellent agreement level in scoring the pre-treatment PAR. Post-treatment assessment shows a moderate agreement level in both DHC and PAR with ICC 0.661 and 0.595 respectively. Although scoring the AC had poor level of agreement among the residents, good reliability was detected among the residents in scoring the ICON index at T2 (ICC = 0.826). The inter-examiner reliability between orthodontic residents and undergraduate students indicates a moderate agreement level in scoring pre-treatment DHC, AC and ICON. Pre-treatment PAR index had a moderate to excellent reliability level among the raters. In post-treatment assessment, the DHC had good reliability while post treatment ICON and the PAR had poor reliability among the raters.

## Discussion

The main findings of the present study were that the ICON index had the best reliability among orthodontic residents in scoring both pre- and post-treatment dental casts. It was also shown that all cases (*n* = 87) had a progressive reduction in post-treatment assessment for all three indices. This was expected due to the active orthodontic treatment that was done.

Previous studies have reported a moderate correlation between AC and DHC of the IOTN, as well as between AC and ICON, in assessing orthodontic treatment need [14], which is consistent with the moderate correlation observed between AC and DHC at T1 in the present study. In addition, our findings demonstrated a high level of correlation between AC and ICON in treatment need assessment. Previous studies concluded that adjusting the cut-off points of AC and ICON may enhance the accuracy of orthodontic treatment need evaluation [22]. Although the PAR index was originally developed as an outcome assessment tool, subsequent studies have documented its usefulness in predicting treatment need [23, 24]. However, a Swedish study reported that PAR is not as good as the Swedish Medical Board Index in estimating treatment need [17]. In contrast, our results showed that PAR exhibited moderate to high correlation with other indices in assessing treatment need.

The present study demonstrated a negligible correlation between AC and DHC in assessing treatment outcome. This discrepancy may be related to the fact that the IOTN was originally developed to assess treatment need rather than treatment outcome. However, an earlier study suggested that the IOTN could also be used to assess treatment outcomes when both pre- and post-treatment casts are evaluated [6]. In the present study, the high correlation between ICON and AC is in line with previous findings showing a strong relationship between these indices when assessing treatment need. This has led some authors to suggest that the IOTN AC may be used as an alternative to ICON for treatment need assessment [14, 25, 26].

Our data indicate low correlation between ICON and DHC, whereas prior research demonstrated strong correlation between ICON and DHC on treatment necessity and outcome evaluation [14]. Another study has also suggested that ICON could be a substitute for IOTN and PAR when assessing the necessity for orthodontic treatment [26, 27].

Since AC is an integral and heavily weighted component of ICON [2], the strong correlation between AC and ICON is expected and should not be interpreted as an independent finding. The lower post-treatment ICON reliability among less experienced examiners may partly be due to the subjective nature of the AC, but the present study was not designed to determine which individual ICON components contributed to the disagreement observed. The subjective nature of both AC and ICON has raised concerns about their sensitivity to detect the need for treatment [28]. However, another study demonstrated that AC and ICON are comparable to other indices in determining treatment need and have good sensitivity [22]. Moreover, a close relationship between PAR and ICON has been reported in several investigations, in line with the results of the present study [27, 29, 30]. However, low to moderate correlations were observed between PAR and the DHC and AC components of the IOTN, respectively.

Multiple studies have examined the reliability of the IOTN, ICON, and PAR indices [22, 31]. Our results are consistent with previous studies showing that both IOTN components are valid and reproducible, with the DHC demonstrating particularly high reproducibility compared with Angle’s classification method [5]. Brook and Shaw also confirmed the reproducibility of the DHC under ideal clinical conditions [16]. Acceptable reliability of the AC has also been reported, with evidence that examiner calibration and repeated use improve scoring consistency over time [32, 33]. ICON has been proven to be a valid and reliable index for assessing orthodontic treatment need, demonstrating moderate to good reliability as well as high sensitivity and specificity [20, 24]. It has also been shown to be a valid tool in evaluating treatment complexity and outcome [21]. A good reliability of ICON in assessing the treatment outcome between the residents agrees with most reproducibility studies of ICON.

The reduced reliability of ICON scoring at T2 among all raters in this study may be explained by differences in orthodontic clinical experience, as well as the heavy weighting of aesthetic assessment within ICON, which introduces subjectivity and may limit reliability [21]. In contrast, PAR has consistently demonstrated excellent reliability [18], a finding supported by our results among both residents and all raters when assessing treatment need rather than outcome.

In terms of feasibility, the mean time for scoring all three indices was recorded. PAR required the longest scoring time. The undergraduate students took an average of 2 minutes for IOTN, 5 minutes for ICON, and 7.5 minutes for PAR, while orthodontic residents required approximately 1, 3, and 5 minutes, respectively.

Several limitations should be acknowledged, including the modification that was made by considering categories of the ICON index (minimally improved and not improved or worse) and (substantially improved and moderately improved) as two wide categories. The ICON index originally had five improvement grades, but the PAR index has three grades after treatment that made the comparison difficult. This modification was made because the sample size was relatively small and certain categories were underrepresented. Moreover, the main research emphasis was on differentiating between clinically significant success and insufficient improvement, rather than assessing fine gradations of change. Further limitation includes the lack of simultaneous calibration among all examiners, and the reduction in sample size following the application of exclusion criteria.

## Conclusions

The hypothesis was partly rejected since the results obtained from this assessment support the following conclusions: a negligible correlation was found between DHC and AC of the IOTN, while low correlations were observed between DHC and both ICON and PAR in assessing treatment outcome. In between the other indices, namely AC, ICON and PAR, a moderate to high correlation was found in assessing the treatment outcome. The ICON index demonstrated the best reliability among orthodontic residents in scoring both pre- and post-treatment dental casts and required less scoring time than PAR, although IOTN was the fastest index to score. The PAR index had good reliability among all raters before treatment and poor reliability after treatment. Whereas, among all raters combined, the DHC had moderate reliability before treatment and was the index with the best reliability in scoring the treatment outcome.
